# The combination therapy with alfacalcidol and risedronate improves the mechanical property in lumbar spine by affecting the material properties in an ovariectomized rat model of osteoporosis

**DOI:** 10.1186/1471-2474-10-66

**Published:** 2009-06-15

**Authors:** Ayako Shiraishi, Sayaka Miyabe, Takayoshi Nakano, Yukichi Umakoshi, Masako Ito, Masahiko Mihara

**Affiliations:** 1 Product Research Department, Chugai Pharmaceutical Co, Ltd., 1-135 Komakado, Gotemba, Shizuoka, 412-8513, Japan; 2 Division of Materials & Manufacturing Science, Graduate School of Engineering, Osaka University, 2-1, Yamada-Oka, Suita, Osaka 565-0871, Japan; 3 Department of Radiology, Nagasaki University School of Medicine, 1-7-1 Sakamoto, Nagasaki, 852-8501, Japan

## Abstract

**Background:**

We conducted the present study to investigate the therapeutic effects of a combination treatment of alfacalcidol (ALF) and risedronate (RIS) on the bone mechanical properties of bone and calcium (Ca) metabolism using an ovariectomized (OVX) rat model of osteoporosis.

**Methods:**

Female Wistar rats were OVX- or sham-operated at 40 weeks of age. Twelve weeks post-surgery, rats were randomized into seven groups: 1) sham + vehicle, 2) OVX + vehicle, 3) OVX + ALF 0.025 μg/kg/day, 4) OVX + ALF 0.05 μg, 5) OVX + RIS 0.3 mg, 6) OVX + RIS 3.0 mg, 7) OVX + ALF 0.025 μg + RIS 0.3 mg. Each drug was administered orally five times a week for 12 weeks. After treatment, we evaluated the mechanical properties of the lumbar vertebra and femoral midshaft. In the lumbar vertebra, structural and material analyses were performed using micro-computed tomography (micro-CT) and microbeam X-ray diffraction (micro-XRD), respectively. Biochemical markers in serum and urine were also determined.

**Results:**

(1) With respect to improvement in the mechanical strength of the lumbar spine and the femoral midshaft, the combination treatment of ALF and RIS at their sub-therapeutic doses was more effective than each administered as a monotherapy; (2) In the suppression of bone resorption and the amelioration of microstructural parameters, the effects of ALF and RIS were considered to be independent and additive; (3) The improvement of material properties, such as microstructural parameters and the biological apatite (Bap) c-axis orientation, contributed to the reinforcement of spinal strength; and (4) The combination treatment of ALF and RIS normalized urinary Ca excretion, suggesting that this treatment ameliorated the changes in Ca metabolism.

**Conclusion:**

These results demonstrate that the combination treatment of ALF and RIS at their sub-therapeutic doses can improve the mechanical properties of the spine as well as the femur and ameliorate changes in Ca metabolism in an animal model of osteoporosis, suggesting that the combination treatment of ALF and RIS has a therapeutic advantage over each monotherapy for the treatment of osteoporosis.

## Background

The incidence of osteoporosis is increasing rapidly in the elderly population. Osteoporosis is a degenerative disease characterized by reduced bone mass and deterioration of bone microstructures which increases the risk of fracture [1]. Osteoporosis is divided into two types: postmenopausal osteoporosis and senile osteoporosis. The causes of postmenopausal osteoporosis are accelerated bone resorption and systemic calcium (Ca) imbalance due to menopause-induced estrogen deficiency. In contrast, senile osteoporosis is attributed to the age-related reduction of osteogenesis, insufficient Ca intake, reduced Ca absorption, and Ca imbalance due to hyperparathyroidism caused by vitamin D deficiency [2].

Alfacalcidol [1α(OH)D_3_; ALF] is a prodrug of active vitamin D_3_, a Ca-regulating hormone, and is frequently used in several countries to treat osteoporosis. There are some studies that have shown ALF to be effective as an anti-osteoporotic agent [3,4], although high doses of ALF have been associated with adverse drug reactions such as hypercalciuria and hypercalcemia. Ovariectomy increases bone turnover in rats by accelerating resorption and formation of bone, and, because bone resorption is dominant, bone mass eventually decreases. Hence, ovariectomized (OVX) rats have been used widely as an animal model of postmenopausal osteoporosis [5]. Previously, we studied the preventive therapy of administering ALF to rats immediately after ovariectomy and found that ALF sustained or increased bone mass by suppressing bone resorption while maintaining bone formation, and, in addition, facilitating the absorption of Ca in the intestine [6].

Risedronate (RIS) is a third-generation bisphosphonate (sodium risedronate hydrate), and like alendronate (ALN), is a potent bone resorption inhibitor that is used clinically to treat osteoporosis [7]. Recently, it has been reported that, although RIS had a role in mineralization, it did not change bone mass and bone structure markedly [8-10]. It has also been shown that in postmenopausal osteoporosis, three- and five-year treatment with RIS increases the degree and homogeneity of mineralization without producing hypermineralization [11], while Boivin GY et al. have reported that long-term treatment with ALN prolongs the viability of bone structure units (BSU) and prolongs the duration of secondary mineralization [12]. Depending on the bisphosphonate used there is a risk of adverse drug reactions, such as upper gastrointestinal tract bleeding, and complicated prescriptions can lead to low compliance.

Based on the present definition of osteoporosis, both density and quality are important for bone strength [13-15]. Although the definition of bone quality remains controversial, it is thought to encompass both structural and material properties of bone. In recent reports, the biological apatite (Bap) c-axis orientation was analyzed by microbeam X-ray diffraction (micro-XRD) as a useful material parameter for bone quality in evaluating mechanical function, in addition to in vivo stress distribution and bone formation processes [16-18]. BAp c-axis has been revealed to be in close alignment with the direction of collagen fibers [19,20], and moreover, Bap has been shown to crystallize under an anisotropic hexagonal lattice, with the mechanical properties of the BAp crystallite depending on the crystal orientation [21]. According to micro-XRD analysis, preferential alignment of the BAp c-axis varies sensitively depending on the portion, bone shape, and in vivo stress distribution [16]. The preferential degree of BAp c-axis orientation relating to the collagen fiber direction also changes according to bone condition with ageing, bone diseases and severity, bone regeneration, and gene defects, etc. [17]. In spite of a case using neutron scattering, for example, the degree of preferential alignment of the BAp c-axis changes with age due to changes in stress at different times in a human's life, resulting in the local maximum at three years old in femora along the bone longitudinal axis [22]. A more recent micro-XRD study showed a negative correlation between the degree of BAp orientation and the mineral apposition rate (MAR) in rat femora [18]. In a pathological state, for example, the degree of osteoporosis accelerates causing the degree of BAp orientation in mice femora to decrease [23]. Thus, the degree of BAp orientation seems to be one of the important indices in evaluating in vivo stress distribution, nano-scale microstructure and related mechanical functions of the damaged bone, and in diagnosing bone diseases.

The objective of this study was to clarify the effects of ALF and RIS combination treatment at their respective sub-therapeutic doses on ameliorating changes in bone mechanical properties and bone metabolism. Our hypothesis was that the combined treatment of ALF and RIS may allow significant increases in bone strength due to suppression of bone resorption compared with each administered as a monotherapy. To test this hypothesis, aged OVX rats were treated 5 times a week with vehicle, ALF (0.025, 0.05 μg/kg), RIS (0.3, 3 mg/kg), or ALF (0.025 μg/kg) combined with RIS (0.3 mg/kg) for 12 weeks. We assessed the parameters as follows; (1) the mechanical properties in lumbar spine and femoral midshaft; (2), the bone density and BAp axis orientation in the cortical region of lumbar vertebra and microstructural parameters in trabecular bone of lumbar vertebra, and (3) urinary deoxypyridinoline (DPD) excretion as a bone resorption marker and serum and urinary biochemical parameters.

## Methods

### Reagents

ALF, synthesized at Chugai Pharmaceutical Co., Ltd. (Gotemba, Japan), was dissolved in medium-chain triglyceride (MCT) and diluted to given concentrations. Commercially available RIS, purchased from LKT Laboratories, Inc. (Minnesota, USA), was dissolved in sterile physiological saline and diluted to given concentrations. Both solutions were administered orally at a dose of 1 mL/kg body weight.

### Experimental Design

All animal studies were performed according to Chugai Pharmaceutical's ethical guidelines for animal care, and the experimental protocols were approved by the Animal Care Committee of the institution. Eleven-month-old female Wistar-Imamichi rats (n = 55) were purchased from the Imamichi Institute for Animal Reproduction (Ibaraki, Japan). The rats were kept in individual stainless steel wire cages, and were allowed free access to commercial standard rodent chow (CE-2; CLEA Japan, Inc. Tokyo, Japan) and tap water. The bone mineral density (BMD) of lumbar vertebrae (L2-L4) was measured in all rats by in vivo scanning using DXA analysis (DCS-600, Aloka, Japan). Based on their spinal BMD, the rats were divided into seven groups with roughly equal average BMD. In all 7 groups, an incision was made in the skin of the back under etherization. Bilateral ovariectomy was performed in six groups; in the remaining group (the sham-operation group), only the skin incision was made. At 12 weeks after surgery, the spinal BMDs of rats in the OVX groups was measured, and the rats were divided into the following six groups with almost the same average BMD: a OVX-vehicle control (OVX) group, a 0.025 μg/kg ALF-treated (ALF-L) group, a 0.05 μg/kg ALF-treated (ALF-H) group, a 0.3 mg/kg RIS-treated (RIS-L) group, a 3.0 mg/kg RIS-treated (RIS-H) group, and a 0.025 μg/kg ALF and 0.3 mg/kg RIS combination-treated (ALF-L + RIS-L) group. ALF and RIS were administered orally once daily, five times a week. ALF was administered between 8:00 and 10:00, and RIS was administered between 14:00 and 16:00. The body weight of each rat was measured weekly, and the volume of drug or vehicle administered was calculated based on the most recent body weight measurement. At 12 weeks after the administration of drug or vehicle, all rats were necropsied. The rats were fasted for 24 hours after the final administration, and a 24-hour urine sample was collected and stored frozen at -70°C. At the time of necropsy, each rat was etherized, a blood sample was collected from the jugular vein, and the serum was isolated and stored frozen at -70°C. After the blood sample was collected, the rats were bled to death, and the lumbar vertebrae (L4-L5) and bilateral femurs were excised. The fifth lumbar vertebra (L5) and left femur, which were used to measure the mechanical strength, were stored frozen at -70°C, and the other vertebra (L4), which was used to measure the BMD and micro-XRD, was fixed in 70% ethanol.

### Biochemical Analysis

Serum concentrations of Ca and inorganic phosphorus (P) were measured using an autoanalyzer (Hitachi 7170; Hitachi Co., Ltd., Tokyo, Japan). Urinary Ca, P and creatinine (CRE) were also measured using the autoanalyzer. Urinary DPD excretion was measured using an OSTEOLINKS-DPD EIA kit (Sumitomo Seiyaku Biomedical Co., Ltd., Osaka, Japan), and the data were corrected for the urinary CRE concentration.

### Measurement of Mechanical Properties

Using a mechanical strength analyzer (TK-252CC; Muromachi Kikai Co., Ltd., Tokyo, Japan), the mechanical strength of the lumbar vertebra (L5) and left femur was measured using a compression test [24] and a three-point bending test [25], respectively.

For the compression test, the planoparallel surfaces were obtained by removing the cranial and caudal ends of the vertebral specimen. From the vertebral body, a central cylinder with planoparallel ends and a height of approximately 5 mm was obtained. A compression force was applied to the specimen in the cranio-caudal direction using a steel disk at a deformation rate of 2.5 mm/min. The ultimate compressive load (N), the stiffness (N/mm), and the energy (mJ) were calculated as the mechanical properties directly from the load-displacement curve.

For the three-point bending test, the left femur was placed on a special holding device with supports located 12 mm apart. A bending force was applied with the cross head at a speed of 20 mm/min, until a fracture occurred. From the load-displacement curve, the ultimate compressive load (N), the stiffness (N/mm), and the energy (mJ) were obtained.

### Structural Analysis using Micro-computed Tomography (micro-CT)

In order to investigate the additive effects of ALF and RIS combination treatment on the trabeculae in the cancellous tissue of the L5 vertebral body, three-dimensional trabecular analysis was performed by micro-CT (μCT40; Scanco Medical, Zurich, Switzerland). The μCT40 is equipped with a microfocus X-ray tube with a focal spot of 10 μm, producing a fan beam that is detected by a charge-couple device array with a turntable that can be shifted automatically in the axial direction. The filtered 40-kVp X-ray spectrum peaks at 25 keV, allowing excellent bone-versus-marrow contrast. The whole spinal body was scanned in 250 slices (thickness, 13 μm) in the dorsoventral direction. On the original three-dimensional images, morphometric indices were directly determined from the binarized volume of interest (VOI) [26]. Three-dimensional reconstruction of bone was performed using the triangulation algorithm. The volume of trabecular bone (BV, mm^3^) was calculated using tetrahedrons corresponding to the enclosed volume of the triangulated surface. Bone surface area (BS) was calculated using the Marching Cubes method to triangulate the surface of the mineralized bone phase. The total tissue volume (TV, mm^3^) is the volume of the whole examined sample. To compare between samples of different sizes, we normalized values such as the bone volume fraction (BV/TV, %) and trabecular bone surface density (BS/BV, %). Using the original application and the method described by Hildebrand et al, we directly measured the following histomorphometric parameters on three-dimensional images, not using the parallel plate model: trabecular number (Tb.N,/mm), trabecular thickness (Tb.Th, μm), and trabecular separation (Tb.Sp, μm) [27]. Thus, Tb.Th, Tb.Sp, and Tb.N were model-independent indices, and were not biased by eventual deviations of the actual structure.

### Measurement of BMD using Peripheral Quantitative Computed Tomography (pQCT)

**Figure 1:**
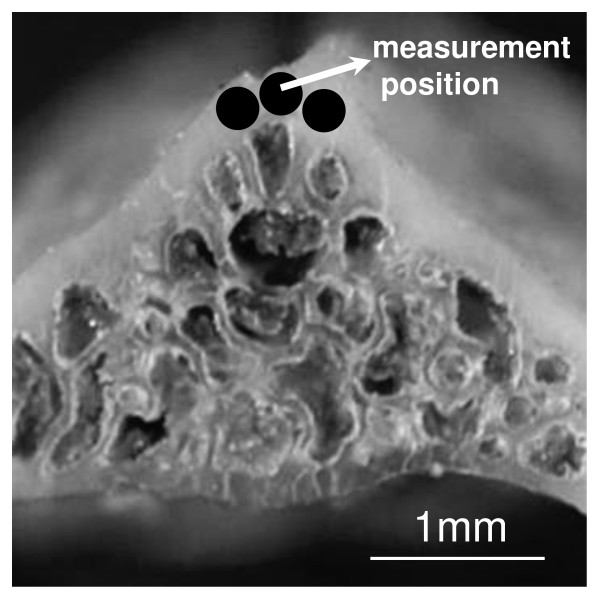
**The regions of pQCT BMD and BAp axis measures in the L4 specimens**.

The BMD (mg/cm^3^) of the ventral cortical portion at the center of L4 was analyzed by the pQCT method using XCT research SA (Stratec Medizintechnik Pforzheim, Germany) [28,29] in a volume of 240 × 240 × 460 μm^3^, where the surface area (240 × 240 μm^2^) corresponded to the same region on which the micro-XRD measurement was performed. Figure 1 shows the regions of pQCT BMD and BAp axis measurements. The volume for each voxel resolution was 80 × 80 × 460 μm^3^.

### Material Analysis using Microbeam X-ray Diffraction (micro-XRD)

Preferential alignment of the BAp c-axis correlating to the collagen arrangement is a useful parameter for evaluating in vivo stress distribution, the process of bone formation and growth, and mechanical function in bone tissues [16]. In order to understand the additive effects of ALF and RIS combination treatment on preferential alignment of the BAp c-axis correlating to the collagen arrangement as a bone material parameter in the cortical portion of the L4 vertebral body, quantitative crystallographic analysis of preferential BAp orientation was performed using a microbeam X-ray diffractometer system (micro-XRD; Mac Science, M18XHF22-SR, Tokyo, Japan). Cu-Kα radiation was generated at a tube voltage of 90 kV and a tube current of 40 mA. Diffracted beams were detected with a curved position sensitive proportional X-ray counter (PSPC). The incident beam was collimated into a circular spot of 50 μm in diameter by a metal collimator, and X-ray diffraction peaks were obtained from a radiated surface area of approximately 250 μm in diameter. The fixed specimen was swung and rotated in the appropriate range of Euler angles to measure the optimal BAp orientation intended in this study. The detailed conditions used in the micro-XRD analysis were as described in a previous paper [16]. Preferential alignment of the BAp c-axis was evaluated as the relative intensity ratio of the (002) diffraction peak to the (310) peak from the X-ray profile measured in the cranio-caudal direction.

The L4 specimens were cut perpendicular to the cranio-caudal axis at the mid point, and the cross sections were abraded by using emery papers of decreasing grit size from no. 600, no. 800, no. 1200 to no. 2000 under water to remove surface damage and roughness until the central part of the vertebral bodies was revealed. The measurements were repeatedly performed three times in each specimen with a slight change in position in the ventral cortical portion on the revealed cross section of L4 (Figure 1).

### Statistical Analysis

All data were expressed as the mean ± standard error (SE). Statistical analysis was performed using analysis of variance (ANOVA) and Statistic Analysis System (SAS) software. The intergroup differences were assessed using the Student's *t *test. To examine the additive effects of ALF and RIS, a two-way ANOVA model of ALF treatment, RIS treatment and interaction between the two drugs was used for the bone mechanical properties, the microstructural parameters, the bone resorption marker, the BAp c-axis orientation and the BMD. The effects of ALF and RIS were considered to be independent and additive, if the interaction effect was not statistically significant (*P *> 0.05) in a two-way ANOVA model, and if the effects of the combination group were significantly higher than the OVX control group and each monotherapy group (*P *< 0.05).

## Results

### 1. Effects on the body weight and the serum and urinary biochemical parameters (Table 1)

**Table 1:** Body weight and biochemical parameters

Group	n	Body weight (g)	Serum Ca (mg/dL)	Serum P (mg/dL)	Urine Ca/CRE	Urine P/CRE
Sham	7	388 ± 14*	9.8 ± 0.2	6.1 ± 0.4	0.095 ± 0.038	1.66 ± 0.29
OVX	8	436 ± 13	10.4 ± 0.3	6.7 ± 0.8	0.144 ± 0.072	1.40 ± 0.13
ALF-L	8	469 ± 11	10.6 ± 0.1	6.7 ± 0.4	0.207 ± 0.031^a)###^	1.73 ± 0.25^#^
ALF-H	8	447 ± 13	10.1 ± 0.4	6.5 ± 0.2*	0.427 ± 0.040^b)^**	2.02 ± 0.21*
RIS-L	8	468 ± 10	9.9 ± 0.3^a)^	5.4 ± 0.2	0.027 ± 0.004^b)^	1.26 ± 0.11
RIS-H	8	459 ± 11	10.2 ± 0.1^b)^	5.4 ± 0.1^a)^	0.027 ± 0.004^b)^	1.31 ± 0.11
ALF-L + RIS-L	8	465 ± 15	10.7 ± 0.1	5.8 ± 0.1	0.132 ± 0.015^##^	1.56 ± 0.14

Ca: calcium, P: inorganic phosphate, CRE: creatinine

Mean ± SE (n = 7–8)

*p < 0.05, **p < 0.01 vs. OVX control group

^a)^p < 0.05, ^b)^p < 0.001 vs. ALF-L+RIS-L group

^#^p < 0.01, ^##^p < 0.01, ^###^p < 0.001 vs. ALF-H group

At the time of necropsy, the average body weight in the OVX group was significantly higher than that of the Sham group (p < 0.05), but the administration of both ALF and RIS did not affect the body weight. Ovariectomy did not significantly alter the Ca and P levels in serum and urine. The serum Ca level in the ALF-L + RIS-L group was significantly higher than that in the RIS-L group or the RIS-H group (p < 0.05, p < 0.01, respectively). The serum P level in the ALF-H group was significantly higher than that in the OVX group (p < 0.05). The serum P level in the RIS-H group was significantly lower than that in the ALF-L + RIS-L group (p < 0.05). ALF increased the Ca excretion in a dose-dependent manner. In contrast, the urinary Ca excretion in the ALF-L + RIS-L group was comparable to that of the Sham group, and there was a significant difference in Ca excretion between the ALF-L + RIS-L group and the ALF-L and the RIS-L groups (p < 0.05 and p < 0.01, respectively). The urinary P excretion in the ALF-H group was significantly higher than that in the OVX group (p < 0.05).

### 2. Effects on the bone resorption marker (Figure 2)

**Figure 2:**
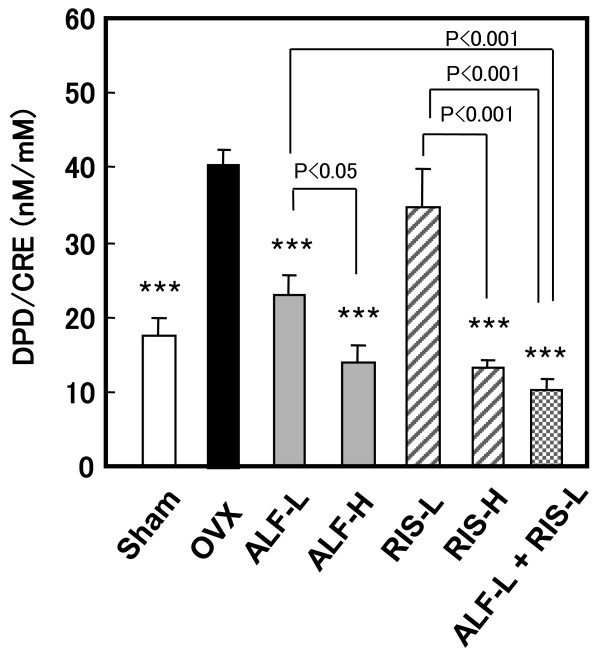
**Effect of alfacalcidol and risedronate, alone or in combination, on the bone resorption marker in OVX rats**. Starting 12 weeks after OVX, drugs were administered orally to rats for 12 weeks. Urinary DPD excretion was measured as biochemical marker of bone resorption, as described in MATERIALS AND METHODS. Data for DPD are corrected for urinary CRE concentrations. Each value represents the mean ± SE (n = 7–8). *p < 0.05, **p < 0.01, ***p < 0.001 compared with the OVX-control group. Note that the combination therapy with ALF and RIS suppressed urinary DPD excretion more effectively than each drug alone. DPD: deoxypyridinoline, CRE: creatinine.

Figure 2 shows the level of urinary DPD excretion, a bone resorption marker. Ovariectomy significantly increased urinary DPD excretion (p < 0.001), while RIS monotherapy decreased the DPD excretion in a dose-dependent manner. There was a significant difference in DPD excretion between the OVX group and the other three groups: the ALF-L, ALF-H, and RIS-L groups (p < 0.001). Moreover, urinary DPD excretion in the ALF-L + RIS-L group was significantly lower than that in the OVX, ALF-L, and RIS-L groups (p < 0.001, p < 0.001 and p < 0.001, respectively). The interaction of ALF and RIS for urinary DPD excretion was not statistically significant (Table 2). Therefore, with respect to the suppression of bone resorption, ALF and RIS were considered to be independent and additive.

**Table 2:** The effects of ALF and RIS on biomechanical properties of bones and urinary DPD excretion

Variable	a two-way ANOVA^1)^		
interaction	ALF-L	RIS-L	ALF × RIS

Urinary DPD excretion	<0.0001	0.0035	0.2115
L5 ultimate load (N)	0.0001	0.3290	0.1776
L5 stiffness (N/mm)	0.0345	0.1031	0.5763
L5 energy (mJ)	0.9173	0.6353	0.3556
Femur ultimate load (N)	0.0979	<0.0001	0.1821
Femur stiffness (N/mm)	0.5386	0.4792	0.6799
Femur energy (mJ)	0.1358	0.0009	0.6119

^1) ^P values were obtained from a two-way ANOVA model.

### 3. Effects on the mechanical properties in the fifth lumbar vertebra (Figure 3)

**Figure 3:**
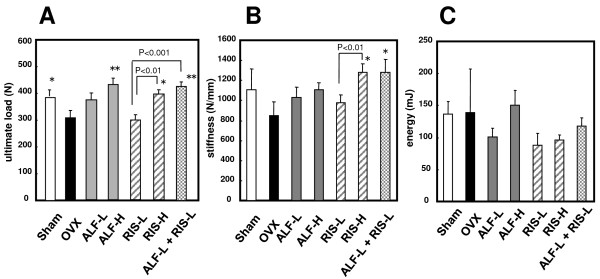
**Effects on the bone mechanical properties of lumbar vertebra in OVX rats**. Starting 12 weeks after OVX, drugs were administered orally to rats for 12 weeks. The ultimate load, the stiffness and the energy in the fifth vertebral body were determined, as described in MATERIALS AND METHODS. Each value represents the mean ± SE (n = 7–8). **p < 0.01, **p < 0.01 compared with the OVX-control group. Note that the combination therapy with ALF and RIS increased the ultimate load more effectively than RIS alone (p < 0.01).

Figures 3A, 3B and 3C show the ultimate load, stiffness, and energy in the lumbar vertebra (L5), respectively.

The ultimate load of the vertebral body in the ALF-H group was significantly higher than that in the OVX group (p < 0.01). In the RIS-H group, the ultimate load was significantly higher than the OVX and RIS-L groups (p < 0.05, p < 0.01, respectively), indicating that RIS monotherapy had a dose-dependent effect on the improvement of the ultimate load and stiffness in the lumbar vertebra. However, in the ALF-L+RIS-L group, the ultimate load of the lumbar vertebra was significantly higher than that in the OVX group or the RIS-L group (p < 0.01, p < 0.001 respectively). The stiffness in the RIS-H group was significantly higher than that in the OVX or RIS-L groups (p < 0.05, p < 0.01, respectively). In the ALF-L + RIS-L group, the stiffness was significantly higher than that in the OVX group (p < 0.05). In contrast, there were no significant differences among each of the groups with respect to energy of the vertebral body.

### 4. Effects on the mechanical properties in the femoral midshaft (Figure 4)

**Figure 4:**
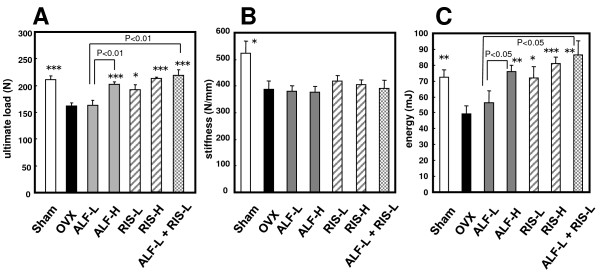
**Effects on the bone mechanical properties of femoral midshaft in OVX rats**. Starting 12 weeks after OVX, drugs were administered orally to rats for 12 weeks. The ultimate load, the stiffness and the energy in the femoral midshaft was determined, as described in MATERIALS AND METHODS. Each value represents the mean ± SE (n = 7–8). *p < 0.05, **p < 0.01, ***p < 0.001 compared with the OVX-control group. Note that the combination therapy with ALF and RIS increased the ultimate load and the energy at femoral midshaft more than ALF alone (p < 0.001).

Figures 4A, 4B and 4C show the ultimate load, stiffness and energy in the femoral midshaft, respectively.

The ultimate load, stiffness and energy of the cortical femur was significantly decreased by ovariectomy (p < 0.001, p < 0.05, and p < 0.01, respectively). ALF monotherapy increased the ultimate load and energy of the femur dose-dependently. In the ALF-H, RIS-L, RIS-H, and ALF-L + RIS-L groups, the ultimate load of the femur was significantly higher than in the OVX group (p < 0.001, p < 0.05, p < 0.001, and p < 0.001, respectively). Furthermore, the ultimate load in the ALF-L group was significantly lower than that in the ALF-L + RIS-L and the ALF-H groups (p < 0.01). The stiffness did not alter with treatment by any of the drugs. The energy of the femur in the ALF-H, RIS-L, RIS-H, and ALF-L+RIS-L groups was significantly higher than in the OVX group (p < 0.01, p < 0.05, p < 0.001, and p < 0.01, respectively). In the ALF-L group, the energy was significantly lower than that in the ALF-H and the ALF-L + RIS-L groups (p < 0.05).

### 5. The microstructural parameters of trabecular bone in the fifth lumbar vertebra by micro-CT analysis (Table 3)

**Table 3:** Microarchitectural indices of lumbar vertebra

Group (/mm)	BV/TV (%)	BS/BV (%)	Tb.Sp (μm)	Tb.Th (μm)	Tb.N
Sham	0.36 ± 0.02^c)^	28.3 ± 0.9	0.124 ± 0.005^c)^	0.071 ± 0.002	5.1 ± 0.1^c)^
OVX	0.26 ± 0.02	31.6 ± 1.6	0.190 ± 0.010	0.064 ± 0.003	4.0 ± 0.1
ALF-L	0.27 ± 0.01**^##^	29.3 ± 0.4*^##^	0.183 ± 0.005**^##^	0.068 ± 0.001*^##^	4.0 ± 0.1**^##^
ALF-H	0.32 ± 0.01^b)^	26.7 ± 0.6^a)^	0.158 ± 0.004^a)^	0.088 ± 0.002^b)^	4.3 ± 0.1^a)^
RIS-L	0.27 ± 0.01*	29.3 ± 0.6*	0.181 ± 0.010	0.068 ± 0.001*	4.0 ± 0.1*
RIS-H	0.29 ± 0.01	28.4 ± 0.5	0.167 ± 0.005	0.071 ± 0.001	4.2 ± 0.1
ALF-L+RIS-L	0.31 ± 0.01^a)^	27.9 ± 0.3^a)^	0.157 ± 0.004^a)^	0.072 ± 0.001^a)^	4.4 ± 0.1^a)^

BV/TV: bone volume, BS/BV: bone surface, Tb.Sp: trabecular separation, Tb.Th: trabecular thickness, Tb.N: trabecular number

Mean ± SE (n = 7–8)

^a)^p < 0.05, ^b)^p < 0.01, ^c)^p < 0.001 vs. OVX control group

*p < 0.05, **p < 0.01 vs. ALF-L+RIS-L group

^#^p < 0.05, ^##^p < 0.01 vs. ALF-H group

Ovariectomy decreased both the bone volume (BV/TV) and trabecular number (Tb.N), and increased the trabecular separation (Tb.Sp), suggesting that ovariectomy caused the cancellous trabecular bone to become thin and sparse, and disrupted the bone structure. Significant improvements, however, were observed in BV/TV, the bone surface (BS/BV), Tb.Sp, trabecular thickness (Tb.Th) and Tb.N in the ALF-H group compared with the OVX group, and RIS monotherapy did not markedly alter any of the examined structural parameters. In contrast, ALF monotherapy dose-dependently ameliorated the structural parameters of trabecular bone in the lumbar spine. In the ALF-L+RIS-L group, there were significant improvements in these microstructural indices compared with the OVX group. Moreover, the BV/TV, BS/BV, Tb.Th and Tb.N in the ALF-L+RIS-L group were significantly higher than those in the ALF-L and the RIS-L groups. The interaction of ALF and RIS for the structural parameters was not statistically significant (Table 4). Therefore, with respect to improvement of the microstructural parameters of trabecular bone, ALF and RIS were considered to be independent and additive.

**Table 4:** The effects of ALF and RIS microstructural indices, 3D-BMD and BAp c-axis orientation in lumbar vertebra

Variable	a two-way ANOVA^1)^		
interaction	ALF-L	RIS-L	ALF × RIS

L5 BV/TV	0.0420	0.0173	0.3994
L5 BS/BV	0.0438	0.0487	0.6317
L5 Tb.N	0.0670	0.0355	0.2750
L5 Tb.Th	0.0352	0.0350	0.8419
L5 Tb.Sp	0.1208	0.0358	0.1493
BAp c-axis orientation	0.2266	0.0599	0.9375
3D-BMD	0.0326	0.8417	0.0020

^1) ^P values were obtained from a two-way ANOVA model.

### 6-1. The material properties in ventral cortical portion of the fourth lumbar vertebra (Figure 5-A, B, C)

**Figure 5:**
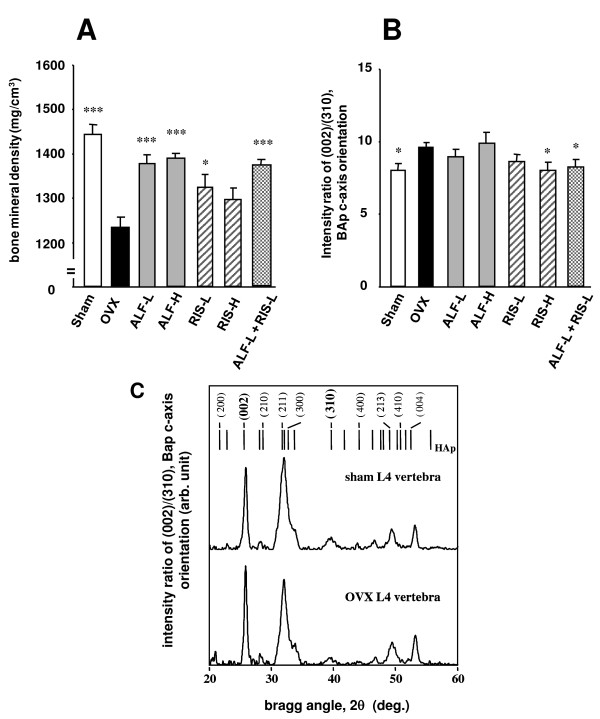
**Effects on the BMD and the BAp orientation at the ventral cortical portion of lumbar spine**. Starting 12 weeks after OVX, drugs were administered orally to rats for 12 weeks. The BMD (A) and the BAp c-axis orientation (B) in the cranio-craudal direction were measured at the ventral cortical portion of the 4^th ^lumbar spine by pQCT and micro-XRD, respectively, as described in MATERIALS AND METHODS. Typical micro-XRD profiles of the OVX and Sham rats were described for clarifying the spectral quality (C). Each value represents the mean ± SE (n = 7–8). *p < 0.05, ***p < 0.001 compared with the OVX-control group. Note that the combination therapy with ALF and RIS recovers the BAp c-axis orientation at the 4^th ^lumbar spine (p < 0.05). Intensity ratio of (002)/(310) corresponds to the orientation degree of the BAp c-axis as a bone quality parameter based on a material property in the cranio-craudal direction. The ratio in randomly oriented apatite powders is approximately 2, and increase in the preferential alignment of the BAp c-axis in the cranio-craudal direction increases the intensity ratio.

As shown in Figure 5-A, the BMD of the restricted cortical volume corresponding to the analyzed portion of the micro-XRD was significantly lower in the OVX group than that in the Sham group (p < 0.001). However, in the ALF-L, ALF-H, ALF-L + RIS-L (p < 0.001) and RIS-L (p < 0.05) groups, the BMD was significantly higher than that in the OVX group (Figure 5-A). Figure 5-B shows the intensity ratio of (002)/(310) corresponding to the orientation degree of the BAp c-axis in the cranio-craudal direction. The ratio in randomly oriented apatite powders (calcium hydroxyapatite; NIST #2910) is about 2, and preferential alignment of the BAp c-axis in the cranio-craudal direction increases the ratio. Figure 5-C shows typical microbeam XRD profiles as a reference. Clear peaks were obtained regardless of whether the bone had low crystallinity. Since the diffraction peaks of (002) and (310) were relatively isolated from the neighboring peaks, a reliable intensity ratio of (002)/(310) can be calculated from the diffraction peaks.

The intensity ratio of (002)/(310) in the OVX group was significantly higher than that in the Sham group (p < 0.05), indicating an increase in the anisotropy of the material parameter, the BAp orientation, by ovariectomy. The increase in intensity ratio of (002)/(310) by ovariectomy was recovered by RIS-H monotherapy and the combination therapy of ALF-L and RIS-L. In other words, ALF/RIS combination therapy restored the bone material parameter, BAp orientation, to the normal state.

### 6-2. Relationship between the BMD and BAp orientation in the cortical portion of the fourth lumbar vertebra (Figure 6)

**Figure 6:**
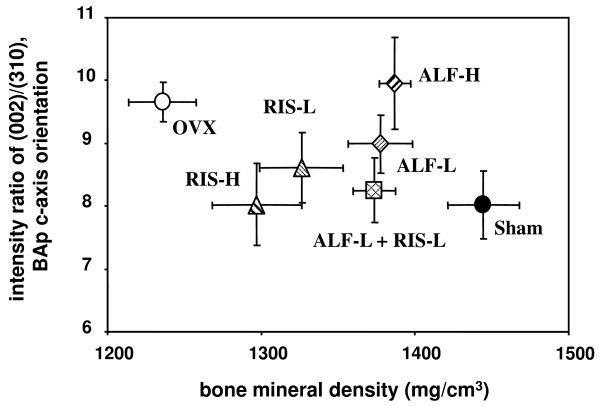
**Effects on balance of BMD and BAp orientation at the ventral cortical portion of lumbar spine**. Starting 12 weeks after OVX, drugs were administered orally to rats for 12 weeks. The BMD and the BAp orientation were measured at the ventral cortical portion of the 4^th ^lumbar spine by pQCT and maicro-XRD, respectively, as described in MATERIALS AND METHODS. Each value represents the mean ± SE (n = 7–8). Note that the combination therapy with ALF and RIS promoted to approach to the normal state of the Sham group. Intensity ratio of (002)/(310) corresponds to the orientation degree of the BAp c-axis as a bone quality parameter based on a material property in the cranio-craudal direction.

BMD and the BAp orientation are independent parameters for describing bone microstructure because they correspond to the density of BAp and the orientation of the BAp c-axis, respectively. Thus, a balance between the two parameters is very important and should be considered. Figure 6 shows the balance between the BMD and the BAp orientation in the ventral cortical portion of a lumbar vertebra (L4) using the results in Figures 5-A and 5-B. The OVX group exhibited lower BMD and higher BAp orientation than the Sham group, and the treated groups were located between the OVX group and the Sham group. Since the normal balance between BMD and BAp orientation corresponds to that in the Sham group, the optimal balance should be approaching that in the Sham group. Higher BAp orientation is not necessarily appropriate and effective for exerting bone mechanical properties under an unpredictable fluctuating load because the optimal degree of BAp orientation must be close to that of the Sham group. The combination therapy of ALF+RIS showed the closest balance of BMD/BAp orientation, indicating the optimal state of the bone material parameters based on the BMD and the BAp c-axis orientation.

## Discussion

In this study, we clearly demonstrated that the combination treatment of ALF and RIS administered at their respective sub-therapeutic doses improved the mechanical properties of the lumbar spine and femoral midshaft in an OVX rat model of osteoporosis by suppression of bone resorption. The improvement of the material properties, such as the microstructural parameters and also the BAp c-axis orientation, contributed to the reinforcement of mechanical strength in the lumbar spine. Moreover, the combination treatment of ALF and RIS normalized the urinary Ca excretion, indicating that this treatment ameliorated the changes in Ca metabolism. These findings suggest that the combination treatment of ALF and RIS had a therapeutic advantage over either the ALF or RIS monotherapy for treating osteoporosis.

Active vitamin D drugs, including ALF, are used to treat osteoporosis due to their safety and ease of administration, although higher doses of these agents can cause hypercalciuria or hypercalcemia. In this study, a high dose of ALF increased the ultimate load of the lumbar vertebra and the ultimate load as well as the energy in the femoral midshaft. The results of detailed micro-CT, micro-XRD and pQCT analyses indicated that the increase in mechanical strength of the lumbar vertebra after treatment with a high dose of ALF was primarily due to the restoration of trabecular bone and the increase in bone density of cortical tissue, whereas ALF did not alter the BAp orientation in the cortical portion as a candidate of bone quality indices. These findings demonstrated that ALF monotherapy improved the mechanical strength of bone by affecting the density and structural factors. We also observed in the present study that ALF dose-dependently increased urinary Ca excretion, as a reflection of intestinal Ca absorption, suggesting that ALF monotherapy caused the hypercalciuria.

It has been reported that bisphosphonates increase bone mass by suppressing bone resorption specifically by inducing apoptosis of osteoclasts [30]. Bisphosphonates that contain nitrogen, such as RIS and ALN, significantly reduce the risk of vertebral and nonvertebral fractures in patients with osteoporosis [31,32]. However, the clinical use of bisphosphonates is rather complicated and the compliance can be low because they are absorbed through the intestinal tract at extremely low rates and give rise to gastrointestinal tract disorders. Also, the mechanisms by which bisphosphonates reduce fractures are not fully understood. Bone quality can be defined as the sum of all factors mediating mechanical competence at constant bone mass. It includes factors such as bone mineral and matrix tissue properties defining mineralization, turnover, and microdamage, as well as bone geometry, mass distribution and microarchitecture [33]. Borah B et al. reported that RIS restored the degree of mineralization and the ratios of low- to highly-mineralized bone to premenopausal levels after three years of treatment and that the BV/TV and trabecular architecture examined by micro-CT analysis did not change from baseline after up to five years of RIS treatment [34]. It was also reported by Fratzl P et al. that in postmenopausal osteoporosis the calcium/vitamin D supplementation increased matrix mineralization (without affecting BV/TV) irrespective of three-year treatment with RIS, suggesting that patients who had low matrix mineralization at baseline had large increases in matrix mineralization density due to calcium/vitamin D supplementation [35].

In the present study, a high dose of RIS increased the ultimate load and energy in the femoral midshaft. This was consistent with micro-XRD examination of the vertebral bodies which showed that the BAp c-axis orientation (which is related to the mechanical function in the cortical portion of L4) recovered with RIS dosage. However, RIS had no clear effects on the microstructural parameters in the trabecular region of the vertebra. For 12 months, Boyce RW et al. repeatedly administered subcutaneous injections of RIS at 5 mg/kg/day for 1 week to OVX rats and then did not administer the drug for the next three weeks beginning when the rats were 3-months-old. They found that the number of trabeculae and their connectivity were significantly increased by this regimen [36]. The differences between the two studies are probably due to the regimens by which the drug was administered. Erben RG et al. investigated the preventive effects of combination RIS/calcitriol (1α25(OH)D_3_) therapy by administering the drugs to 4-month-old rats immediately after ovariectomy [37]. They had reported that, when these drugs were administered at monotherapeutic doses previously shown to suppress the decreases in bone mass and mechanical strength, they further increased the bone mass and mechanical strength of lumbar vertebrae and long bones. Harris ST et al. found in a clinical study that RIS therapy combined with HRT (which suppresses bone resorption) increases the non-vertebral BMD more markedly than it increases the vertebral BMD, and suppresses bone turnover, as indicated by markers of bone metabolism [38]. However, in a recent clinical trial of the effects of a combination treatment of ALN and recombinant human PTH (1–84) (a bone formation accelerator) on BMD, some dose combinations had negligible pharmacological effects [39]. This indicated that not all combination therapies are additive toward bone loss. Therefore, it is necessary to identify the effective combinations and administration methods not only on bone density but also bone quality in osteoporosis treatment.

The analysis of bone metabolic markers revealed that, although ovariectomy accelerated urinary DPD excretion, the combination therapy of ALF and RIS significantly lowered DPD excretion compared to the ALF-L or RIS-L monotherapy, and the interaction was not statistically significant. It suggested that ALF and RIS suppressed bone resorption independently and additively. In the lumbar vertebra, both ALF-L and RIS-L did not significantly alter the ultimate load and stiffness compared with the OVX control group. However, in the combination group, both the ultimate load and the stiffness were significantly higher than the OVX control group. Also, in the femoral midshaft, the ultimate load and energy in the combination group were significantly higher than the OVX control group, whereas those in the ALF-L group or the RIS-L group were not different from the OVX control group. Furthermore, in the improvement of the microstructural indices, ALF and RIS were also considered to be independent and additive.

In this study, the BMD and BAp axis orientation at ventral cortical position and the microstructural parameters at trabecular region were assessed to determine a candidate for the spinal strength improvement. The BMD was significantly higher in the ALF-L group or the RIS-L group as well as the combination group compared with the OVX control group. In contrast, the BAp c-axis orientation in the combination group was significantly lower than the OVX control group, although those in both the ALF-L group and the RIS-L group were not changed compared with the OVX group. Therefore, in the present study, it seems that the material properties, such as the microstructural parameters and the BAp c-axis orientation, contributed to the reinforcement of spinal strength.

In patients with senile osteoporosis, Ca deficiency caused by low absorption of Ca may be a problem. Although this Ca deficiency can be corrected by active vitamin D administration due to facilitation of Ca absorption through the gastrointestinal tract, such treatment may increase the risk of hypercalciuria and hypercalcemia. Bisphosphonates may cause hypocalcemia by suppression of bone resorption. It also has been reported that bisphosphonates are cytotoxic to human colon cancer cells, which have been used in an intestinal epithelial model [40], and thus may affect Ca channel function. The different mechanisms of these drugs on bone metabolism suggest that a combination therapy using an active vitamin D agent with a bisphosphonate could be particularly effective at reducing bone loss and simultaneously improving Ca homeostasis in osteoporosis. Therefore, it is necessary to compare the effects on Ca absorption and excretion between the ALF + RIS combination therapy and each monotherapy. In this study, it was observed that the dose-dependent promotion of urinary Ca excretion by ALF monotherapy was canceled by the combination treatment with RIS, and consequently urinary Ca excretion was maintained at a normal level. Taken together, the simultaneous administration of ALF and RIS at their sub-therapeutic doses improves the Ca balance, suggesting that the combination therapy at lower dose may reduce the risk of known adverse drug reactions by these two drugs alone.

The aim of this study was to determine whether the additive effects of ALF and RIS combination therapy have advantages over each monotherapy. Based on our results, we concluded that: (1) with respect to the improvement of the mechanical strength of lumbar spine as well as the femoral midshaft, the combination treatment of ALF and RIS at their sub-therapeutic doses was more effective than the each monotherapy; (2) in the suppression of bore resorption and the amelioration of microstructural parameters, the effects of ALF and RIS were considered to be independent and additive; (3) the improvement of material properties, such as the microstructural parameters and the BAp c-axis orientation, contributed to the reinforcement of spinal strength; and (4) the combined therapy of ALF and RIS normalized the urinary Ca excretion, suggesting that this therapy ameliorated the changes in Ca metabolism.

## Conclusion

These results demonstrate that the combined ALF and RIS therapy at their sub-therapeutic doses can improve the mechanical properties of the spine and femur and ameliorate Ca metabolism in an animal model of osteoporosis, suggesting that the combination therapy of ALF and RIS has a therapeutic advantage over each monotherapy for the treatment of osteoporosis.

## Abbreviations

ALF: alfacalcidol; RIS: risedronate; OVX: ovariectomized; micro-CT: Micro-computed Tomography; micro-XRD: microbeam X-ray diffraction; BAp: biological apatite; pQCT: peripheral quantitative computed tomography.

## Competing interests

This research project was fully funded by Chugai Pharmaceutical Co., Ltd. AS and MM are research scientists in Chugai Pharmaceutical Co., Ltd. AS carried out the animal studies, conceived of this study and drafted the manuscript. MM participated in the design of this study and helped to draft the manuscript. Alfacalcidol is a product of Chugai Pharmaceutical Co., Ltd. The results from this study may be beneficial for the use of alfacalcidol in the treatment of osteoporosis.

## Authors' contributions

AS carried out the animal studies, conceived of the study and drafted the manuscript. SM, TN and YU performed pQCT and micro-XRD analyses, and provided useful discussions. MI carried out the micro-CT analysis and provided useful discussions about the results. MM participated in the design of this study and helped to draft the manuscript. All authors read and approved the final manuscript.

## Pre-publication history

The pre-publication history for this paper can be accessed here:


